# Agouti Revisited: Transcript Quantification of the ASIP Gene in Bovine Tissues Related to Protein Expression and Localization

**DOI:** 10.1371/journal.pone.0035282

**Published:** 2012-04-17

**Authors:** Elke Albrecht, Katrin Komolka, Judith Kuzinski, Steffen Maak

**Affiliations:** Research Unit Muscle Biology and Growth, Leibniz Institute for Farm Animal Biology (FBN), Dummerstorf, Germany; Deakin School of Medicine, Australia

## Abstract

Beside its role in melanogenesis, the agouti signaling protein (ASIP) has been related to obesity. The potentially crucial role in adipocyte development makes it a tempting candidate for economic relevant, fat related traits in farm animals. The objective of our study was to characterize the mRNA expression of different ASIP transcripts and of putative targets in different bovine tissues, as well as to study consequences on protein abundance and localization. ASIP mRNA abundance was determined by RT-qPCR in adipose and further tissues of cattle representing different breeds and crosses. ASIP mRNA was up-regulated more than 9-fold in intramuscular fat of Japanese Black cattle compared to Holstein (p<0.001). Further analyses revealed that a transposon-derived transcript was solely responsible for the increased ASIP mRNA abundance. This transcript was observed in single individuals of different breeds indicating a wide spread occurrence of this insertion at the ASIP locus in cattle. The protein was detected in different adipose tissues, skin, lung and liver, but not in skeletal muscle by Western blot with a bovine-specific ASIP antibody. However, the protein abundance was not related to the observed ASIP mRNA over-expression. Immuno-histochemical analyses revealed a putative nuclear localization of ASIP additionally to the expected cytosolic signal in different cell types. The expression of melanocortin receptors (MCR) 1 to 5 as potential targets for ASIP was analyzed by RT-PCR in subcutaneous fat. Only MC1R and MC4R were detected indicating a similar receptor expression like in human adipose tissue. Our results provide evidence for a widespread expression of ASIP in bovine tissues at mRNA and, for the first time, at protein level. ASIP protein is detectable in adipocytes as well as in further cells of adipose tissue. We generated a basis for a more detailed investigation of ASIP function in peripheral tissues of various mammalian species.

## Introduction

The *agouti* locus (agouti signaling protein, ASIP) was the first obesity gene to be cloned in mice [1], [2]. Under physiological conditions however, its expression is restricted to hair follicles in mice and influences pigment production in melanocytes by antagonizing the melanocortin-1 receptor (MC1R). By this binding, the α-melanocyte stimulating hormone (α-MSH) mediated synthesis of cyclic adenosine monophosphate (cAMP) is inhibited [3]. The production of black-brown (eumelanin) pigment is then shifted to yellow-red (pheomelanin) pigment (reviewed by Cone et al. [4]). This general mechanism is involved in skin pigmentation of mammalian species as well as in feather pigmentation of birds (e.g. [5], [6]).

Michaud et al. [7] identified a fusion transcript of ASIP and a non-coding exon of an adjacent gene (RALY, hnRNP protein associated with lethal yellow) as cause for embryonic lethality in mice homozygous for this aberration. Heterozygotes display an ectopic expression of ASIP resulting in obesity, insulin resistance, and increased tumor susceptibility, beside yellow coat color [8], [9].

In contrast, ASIP is expressed in a wide variety of tissues in other mammals (human, cattle, and rabbit) as well as in chicken adipose tissue without any pathological signs [6], [10]–[15]. Since human ASIP is mainly expressed in adipose tissue, a role of this protein in the development of metabolic disorders like obesity and insulin resistance was assumed early [10], [16]. It was shown that human ASIP antagonizes different members of the melanocortin receptor (MCR) family in vitro [17]. However, it is still not fully clear which receptor is the target of ASIP in different tissues [18]–[20]. Furthermore, Xue and colleagues [16] reported an inhibition of the Ca^2+^ - dependent lipolysis in human adipocytes in a MCR independent manner, and Mynatt and Stephens [21] proposed a regulation of the adipocyte metabolism by ASIP through influencing the expression of STAT 1/3 (signal transducer and activator of transcription) and PPARG (peroxisome proliferator-activated receptor gamma). Smith et al. [19] developed a working model with an increased ASIP transcription in response to cortisol and assumed effects on both adipocyte differentiation and proliferation. They concluded that ASIP acts as a paracrine factor in the regulation of adiposity, either during development or later in life.

The potentially crucial role of ASIP in adipocyte development makes it a tempting candidate for economic relevant, fat related traits in domestic animals. Sumida et al. [11] first demonstrated ASIP mRNA expression in subcutaneous adipocytes of cattle. Several transcripts of the gene were described in cattle, comprising different non-coding exons with identical coding sequences [6]. This group found an insertion of a bovine full-length long interspersed element (LINE, L1-BT) in the genome of French Normande cattle. A part of the internal promoter of this L1-BT is incorporated as a non-coding exon in an ASIP transcript and leads to over-expression of the gene in several tissues. This transcript was furthermore identified in skin and assumed as causal variant for the brindle coat color of Normande cattle. A potential role of this transcript for meat and milk production was proposed [12]. However, to our knowledge, investigations into the role of ASIP in traits beside coat color in domestic animals are limited to a single study on ASIP mRNA expression in adipose tissue of chicken demonstrating a decreased expression in response to 48 hours of fasting [14].

In a previous experiment we compared morphological and physiological parameters in Japanese Black (JB) and Holstein (HS) steers fed a high-energy diet. The average intramuscular fat (IMF) content in M. longissimus was 34.3±4.2% in JB and 20.4±5.5% in HS at slaughter with 26 months of age [22]. An analysis of gene expression profiles in M. longissimus of each three animals of both breeds revealed ASIP mRNA expression in JB but not or at marginal level in HS muscle, respectively (Maak, unpublished data). We thus hypothesized that differential ASIP expression may contribute to large differences in IMF content observed between different cattle breeds. The objective of our study was to characterize the mRNA expression of different transcripts of the ASIP gene and of putative target molecules in bovine adipose tissues. Furthermore, we investigated the consequences on bovine ASIP protein abundance and localized the protein at cellular level in different tissues.

## Materials and Methods

### Animals and samples

Animal experiments were described by Albrecht et al. [22] and Gotoh et al. [23]. In the current study we used tissue samples of 6 Japanese Black, 5 Holstein and 6 Charolais steers collected at regular slaughter of the animals. Samples of M. longissimus (MLD), intramuscular fat (IMF), intermuscular fat (IMRF) and subcutaneous fat (SCF) were available for RNA and protein isolation. Furthermore, 5 crossbred bulls from a commercial farm were sampled at regular slaughter. Details on the sampled tissues are given in Table 1. Animal care and tissue collection procedures followed the guidelines of the German Law of Animal Protection and the experimental protocol was approved by the Animal Care Committee of the State Mecklenburg-Western Pomerania, Germany (State Office for Agriculture, Food Safety and Fishery; LALLF M-V/TSD/7221.3-2.1-010/03). Animals were slaughtered at the slaughterhouse of the Leibniz Institute for Farm Animal Biology (FBN) Dummerstorf, Germany (EEC Approval Number ES1635/EZ1635) or at E. Faerber GmbH Grossschlaechterei & Co. Belgern, Germany (Crossbred bulls; EEC Approval Number ES729/EZ729/EV729). Samples for protein and DNA isolation were shock-frozen in liquid nitrogen and stored at −80°C. Tissue samples for RNA analysis were immediately transferred to RNAlater (Applied Biosystems, Darmstadt, Germany) and stored at −80°C until further processing. Additionally, 23 DNA samples of bulls from 5 different breeds and crosses were available from a DNA repository at the institute (Table S1).

**Table 1:** Characterization of animals and number of tissue and DNA samples.

			Number of samples								
Breed/Cross	Age (mo)	IMF (%)	DNA	MLD	IMF	IRMF	SCF	Skin	Heart	Liver	Lung
Japanese Black (JB)	26	34.3±4.2	6	6	5	6	6	-	-	-	-
Holstein (HS)	26	20.4±5.5	5	5	5	5	5	-	-	-	-
Charolais (CH)	26	6.4±1.8	6	6	6	6	6	-	-	-	-
Crossbred (JB x diverse; CB)	15	2.5±0.5	5	5	-	5	5	5	5	5	4

MLD: M. longissimus, IMF: intramuscular fat, IRMF: intermuscular fat, SCF: subcutaneous fat.

### DNA and RNA isolation, cDNA synthesis

DNA was extracted from 30 mg liver or muscle tissue by phenol chlorophorm isopropanol precipitation with proteinase K treatment according to standard procedures. For RNA isolation, adipose tissues were homogenised in Qiazol lysis reagent (Qiagen, Hilden, Germany) with a Polytron homogenizer (Kinematica AG, Littau-Luzern, Switzerland) and total RNA was extracted with the RNeasy Lipid Tissue Mini Kit (Qiagen, Hilden, Germany) according to the manufacturer's instructions. Muscle tissue was homogenized using the Xiril Dispomix (Xiril, Hombrechtikon, Switzerland) and Qiazol Lysis Reagent (Qiagen, Hilden, Germany) as described by the manufacturer. The RNA was isolated and purified with NucleoSpin Extract II reagent (Macherey-Nagel, Dueren, Germany) according to manufacturer's guidelines. RNA was quantified with a NanoDrop ND-1000 spectrophotometer (Peqlab, Erlangen, Germany). The RNA integrity was determined with an Experion Automated Electrophoresis System using the RNA StdSens analysis chip (Bio-Rad, Munich, Germany). First strand cDNA was synthesized from 100 ng total RNA of the respective tissue in 20 µl reaction volume using iScript cDNA Synthesis Kit (Bio-Rad, Munich, Germany) according to the provided protocol.

### cDNA-PCR

Qualitative detection of ASIP transcripts in different tissues and of MCR1 to 5 in SCF was performed in a cDNA-PCR approach. We designed specific primers (Table S2) with the software Primer 3 (v. 0.4.0., http://frodo.wi.mit.edu/primer3/) and amplified the target sequences in a 25 µl reaction volume containing 10 ng cDNA, 2 µM of the respective primer pair, and PCR master mix (2×) (Fermentas, St. Leon-Rot, Germany) in a pecSTAR 96 Universal thermocycler (Peqlab, Erlangen, Germany). The amplification followed a standard PCR protocol (initial denaturation 94°C for 4 min, 40 cycles with 94°C for 30 s, template specific annealing temperature for 30 s, 72°C for 30 s, and final step 72°C for 7 min). The annealing temperatures are given in Table S1. The amplicons were subjected to electrophoresis on 3.0% agarose gels containing ethidium bromide and visualized under UV light. In all experiments negative controls (H_2_O instead of cDNA) were used. For the intronless genes for MC1R, 3R, 4R and 5R and for MC2R we used a genomic DNA sample as positive amplification control.

### Quantitative PCR (qPCR)

We used the iCycler MyiQ 2 with iQ detection system (Bio-Rad, Munich, Germany) for qPCR. The gene expression measurement was performed in triplicates in 10 µl reaction volumes containing 10 ng cDNA template, 2 µM of the respective forward and reverse primers and 5 µl SYBR Green Supermix (Bio-Rad, Munich, Germany). The primers listed in Table S1 were designed with Primer 3 (Version 0.4.0., http://frodo.wi.mit.edu/primer3/) and synthesized by Sigma-Aldrich (Munich, Germany). The amplification involved a denaturation step (95°C for 3 min) followed by 45 cycles (95°C for 10 s, 60°C for 30 s, 70°C for 45 s). The specificity of the amplicons was analyzed by melting curve analysis. C_p_ values were determined automatically by iQ5 Software (Version 2.1.97.1001, Bio-Rad, Munich, Germany). For each qPCR the amplification efficiency was calculated from a standard curve derived from six serial dilutions (1∶1, 1∶10, 1∶50, 1∶100, 1∶500, 1∶1,000). The efficiency E was calculated as E = 10^−1/slope of standard curve^−1. The identity of the products was confirmed by sequencing (ABI PRISM 310 Genetic Analyzer; Applied Biosystems, Darmstadt, Germany). Results are expressed as fold-changes in experimental groups compared to a control group with 95% confidence intervals. Holstein was used as control group. The expression values were normalized to beta-2-microglobulin (B2M) and ubiquitously-expressed transcript (UXT) [24] using the efficiency-corrected ΔΔ-C_P_ method described by Pfaffl et al. [25]. The significance of expression differences was calculated with the REST algorithm (REST 2009, Version 2.0.13, QIAGEN, Hilden, Germany).

### Bovine ASIP antibody

A custom made bovine ASIP specific, polyclonal antibody was generated in rabbit (Thermo Fisher Scientific, Huntsville, USA). Two rabbits were immunized with a 14 amino acids long synthetic peptide (aa 24 to 37 of bovine ASIP, Q29414 UniProtKB) coupled to KLH (keyhole limpet hemocyanin) as carrier protein. The standard 70-days immunization protocol was extended for one animal to increase the affinity maturation of the antibody and to increase the immune response from the animal. Finally, the antibody was affinity-purified against the peptide antigen and had a concentration of 2.3 mg/ml.

### Western blotting

Total protein was extracted using CelLytic MT lyses reagent (Sigma-Aldrich, Munich, Germany) with protease inhibitor according to manufacturer's instructions. Protein extract, 40 µg, was mixed with loading buffer and denatured by boiling for 5 min before loading on a 15% SDS-PAGE 10×10 cm mini gel. One or two molecular weight markers were used to determine the molecular weight of the protein bands (Page Ruler and Molecular Weight, Fermentas). After electrophoresis (Peqlab, Erlangen, Germany), proteins were transferred to a polyvinylidene difluoride (PVDF) membrane (Carl Roth, Karlsruhe, Germany) using a semi dry blotter (Biotec-Fischer, Reiskirchen, Germany). The membrane was blocked with 5% non-fat dry milk in Tris-buffered saline (TBS) for 1 h at room temperature. The membranes were than incubated with the described custom made polyclonal antibody against bovine ASIP (Thermo Fisher Scientific, Huntsville, USA) at 4°C overnight, dilution 1∶10,000 of the affinity purified antibody. As a control for specific binding of the antibody, a second membrane was processed in parallel, which was incubated with the antibody previously blocked by the respective peptide. After washing, membranes were incubated with the respective HRP conjugated secondary antibody, (rabbit IgG TrueBlot, 1∶50,000; 18–8,816, eBioscience, Frankfurt, Germany). Antibody label was detected with chemiluminescence substrate (Super Signal West Femto, Thermo Fisher Scientific, Bonn, Germany) and a Chemocam HR-16 imager (INTAS, Göttingen, Germany).

### Immuno-histochemical analysis

Samples of different tissues were cryo-sectioned (8 µm thick) using a Leica CM3050 S (Leica, Bensheim, Germany) cryostat microtome. Sections were shortly fixed in ice cold acetone for 10 s and air dried. Tissue sections were fixed with 4% paraformaldehyde and washed with PBS. Unspecific binding of the secondary antibody was blocked using 10% goat serum in PBS for 15 min. Sections were incubated with the primary antibody against ASIP (1∶100) for 1 h at room temperature in a humidity chamber. Specific binding of primary antibody was detected with the respective goat anti rabbit IgG secondary antibody labelled with Alexa Fluor 488 (Molecular Probes, Eugene, USA). Nuclei were counterstained with 1 µg/ml Hoechst 33258 (Sigma-Aldrich, Munich, Germany). Slides were covered using MobiGLOW mounting medium (MoBiTec, Goettingen, Germany) and appropriate cover-slips. Negative controls were incubated either omitting the primary antibody or blocking the primary antibody with the respective peptide. No unspecific binding of the secondary antibody and only minimal unspecific binding of the primary antibody was detected. Disturbing autofluorescence, especially in heart tissue, was reduced by including a step of Sudan black (0.1% in 70% Ethanol) staining for 30 min prior to the blocking step. Immunofluorescence was visualized with a Nikon Microphot SA fluorescence microscope (Nikon, Duesseldorf, Germany) and an image analysis system equipped with CELL∧F software and a CC-12 high resolution colour camera (OSIS, Muenster, Germany).

### Genotypes at the bovine MC1R locus

Standard PCR was performed with genomic DNA and the primers MC1R *for* and *rev* (Table S2). The resulting PCR product of 233 bp included the polymorphic sites described by Klungland et al. [26]. After purification, the PCR products were subjected to cycle sequencing and analyzed on an ABI PRISM 310 Genetic Analyzer (Applied Biosystems, Darmstadt, Germany). The PCR primers were used for sequencing. The SNP c.293C>T and c.308G>Del defined the genotypes E or E^D^ and e, respectively [26].

## Results

### ASIP mRNA abundance in intramuscular fat

**Figure 1:**
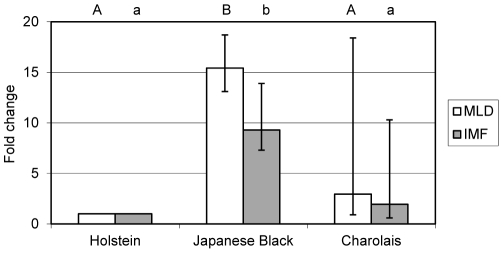
ASIP mRNA abundance in M. longissimus (MLD) and dissected intramuscular fat (IMF). Bars represent means of fold changes compared to Holstein (n = 5) with 95% confidence interval, marked by vertical lines. Different letters indicate significant differences to Holstein within tissue (p<0.05). Number of samples: Japanese Black (n = 6 [MLD], n = 5 [IMF]), Charolais (n = 6).

We employed RT-qPCR to determine ASIP mRNA abundance in M. longissimus containing IMF in JB, HS and CH steers. The amplified fragment included two exons of the coding region (see Table S2 for primers). As expected from the array results, JB animals (n = 6) displayed a robust ASIP expression, whereas the signals in HS samples (n = 5) were close to the detection limit. ASIP mRNA abundance was more than 15-fold higher in JB muscle than in that of HS animals (p<0.001; Figure 1). Charolais steers (n = 6) revealed an unexpected high variation of the mRNA abundance. In four animals RT-qPCR results were similar to that observed in HS but in two CH samples we found an expression of ASIP at the same level like in JB cattle. To account for the high differences in IMF content of the analyzed muscle samples, we measured ASIP mRNA abundance in dissected IMF of all animals of the three breeds separately. The results were similar to that obtained before in muscle (including IMF) with high expression in JB IMF (9-fold higher than in IMF from HS, p<0.001) and heterogeneous expression in the CH samples (Figure 1).

### Expression of different ASIP transcripts in bovine adipose and further tissues

**Figure 2:**
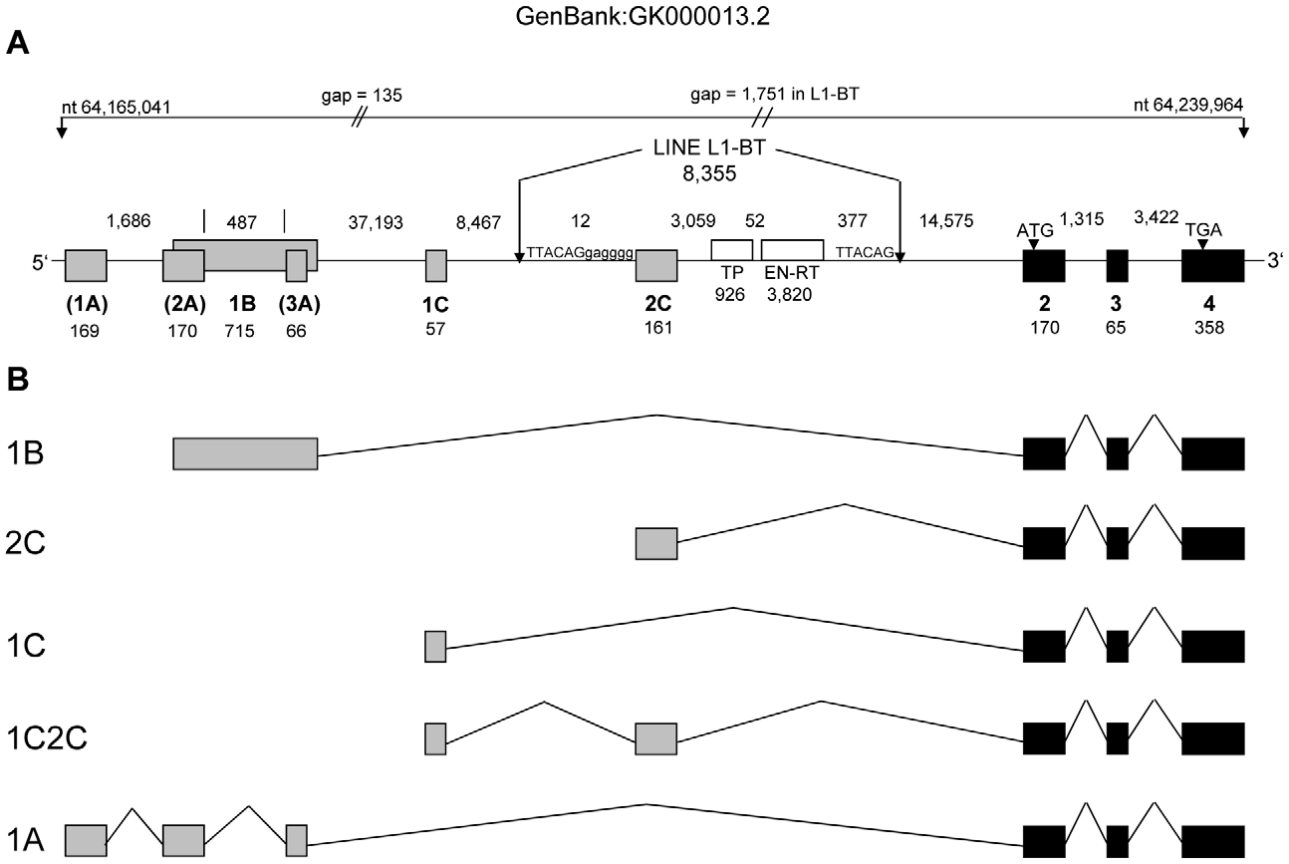
Structure of the bovine ASIP locus and resulting transcripts. (**A**) Non-coding (gray) and coding exons of ASIP (black) are given as boxes and are numbered below. Exons in parentheses were not observed in this study. Smaller numbers indicate exon and intron sizes in base pairs. A LINE element (L1-BT) is inserted between non-coding and coding exons. The underlying sequence (GenBank accession no. GK000013.2) contains two gaps. The size of the first gap was determined by sequencing whereas the second gap was closed in silico by insertion of partial sequence from DQ000238.1. (**B**) Transcripts of the bovine ASIP gene resulting from different use of non-coding exons. Transcript 2C recruits a non-coding exon from the LINE. Transcript 1A was not observed in our study. The figure was modified and supplemented on the basis of data from Girardot et al. [6], [12].

**Figure 3:**
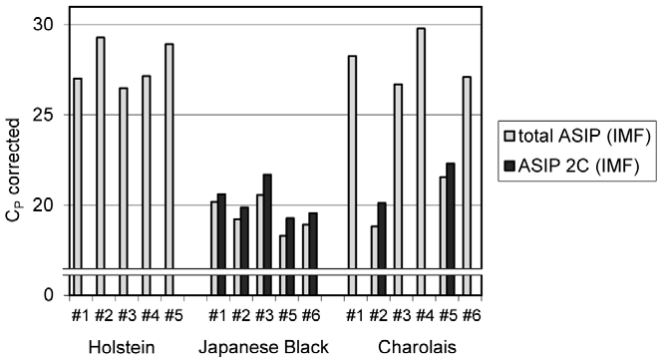
ASIP mRNA abundance (all transcripts) and abundance of transcript 2C in bovine intramuscular fat (IMF). Bars represent individual C_p_ from qPCR in IMF of Holstein, Japanese Black and Charolais steers normalized to 2 reference genes. Note that high ASIP mRNA abundance (low C_p_) was observed in samples with expression of transcript 2C only.

To address the role of different transcripts for mRNA abundance, we searched both available bovine genome assemblies for the ASIP locus and identified a partial LINE sequence with annotated ASIP exon 2C on bovine chromosome 13 in assembly UMD 3.1 [27]. In contrast, ASIP is not yet completely annotated in the current reference sequence of this chromosome (build Btau 5.2). By combining in silico-analysis and DNA sequencing, we closed two remaining sequence gaps at this locus and we established a contiguous genomic sequence of bovine ASIP spanning ∼75 kb and containing all non-coding exons described by Girardot et al. [6], [12] (Figure 2). These non-coding exons were located up to 70 kb upstream the first coding exon. Among the resulting transcripts, one was composed of the partial internal promoter of a bovine LINE L1-BT and the ASIP coding exons. This transcript (named 2C) was considered unique to French Normande and Montbeliarde cattle breeds and it was over-expressed in several tissues [12]. To clarify whether this specific transcript 2C is responsible for the highly variable ASIP expression pattern observed in our analyzed breeds, we designed specific primers and quantified it by RT-qPCR in IMF. We observed an exclusive and high expression of the transcript 2C only in samples of JB and those of CH with a high total ASIP mRNA expression (Figure 3).

The individual ASIP expression pattern in a further adipose depot - subcutaneous fat (SCF) - was identical to that observed in IMF of the corresponding animals as revealed by RT-qPCR analysis (data not shown). Additional analysis of SCF from 5 crossbred bulls (CB; Table 1) revealed four samples with ASIP mRNA abundance comparable to HS but one sample with an expression as high as observed in JB and two CH steers. Again, transcript 2C was exclusively responsible for the increased total ASIP expression in this single CB bull (data not shown). In contrast, the abundance of transcript 1B was similar in SCF of all investigated animals (Figure S1). Non-coding exon 1B was found to be 19 bp longer than that annotated in GenBank (Accession No. DQ000237). Further transcripts were neither detected in IMF nor in SCF.

**Figure 4:**
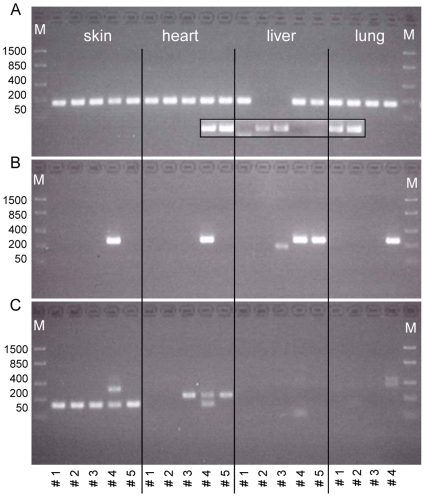
Expression of ASIP transcripts in different tissues of crossbred bulls. All transcripts were amplified in cDNA (40 cycles) from the tissues indicated on panel (**A**). The band intensity is not indicative for mRNA abundance. (**A**) Transcript 1B (157 bp). A repeated amplification is shown in the framed box indicating poor reproducibility in liver cDNA. (**B**) Transcript 2C (244 bp) is detectable in all tissues of bull #4. Sequencing of the product obtained in liver cDNA of bull #5 failed. (**C**) Skin specific transcript 1C (132 bp). The larger band observed in bull #4 was identified as transcript 1C2C (293 bp) by sequencing. M: molecular weight marker.

To characterize the expression pattern of ASIP in cattle, the analyses were extended to further tissues and transcripts. We designed primers specific for all five described transcripts (Figure 2) and performed qualitative RT-PCR analyses in skin, heart, liver, and lung samples of five crossbred bulls used in the previous analyses. We monitored the crossing point (C_p_) of each individual reaction and the transcript specificity was assessed by melting curve analysis. Although this was a qualitative detection procedure, the measured parameters allowed for limited comparison with the quantitative results obtained for M. longissimus, IMF and SCF. Transcript 1B was expressed in skin, heart and lung with similar C_p_ like in the tissues mentioned before (Figure 4). The results obtained with liver cDNA were hardly reproducible, however. Transcript 2C was expressed in all tissues of crossbred bull #4 which was, as shown before, the only animal from this group expressing this specific transcript in adipose tissues. The band obtained for crossbred bull #5 in liver cDNA proved to be unspecific. The transcript 1C was detected as expected in skin cDNA of all animals, but not in other tissues. Transcript 1C2C was co-amplified and confirmed by sequencing in the skin sample of crossbred bull #4 (Figure 4). In this transcript, exon 1C is linked to 2C by six nucleotides (GCGGGG) followed by the coding exons. The heart specific transcript 1A [12] was neither observed in heart nor in any other tissue investigated here.

### ASIP protein abundance in bovine tissues

**Figure 5:**
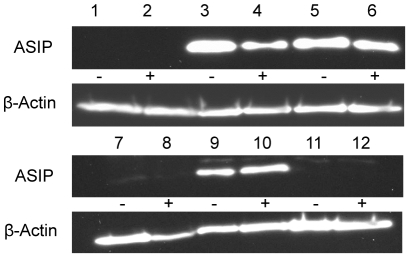
ASIP protein expression in different tissues of bulls with normal expression (−) or over-expression of ASIP mRNA (+). Chemiluminescence detection of ASIP and β-actin by Western blotting of 40 µg protein of the respective tissues. Lanes 1 and 2: M. longissimus, 3 and 4: subcutaneous fat, 5 and 6: intermuscular fat, 7 and 8: heart, 9 and 10: liver, 11 and 12: lung.

ASIP protein abundance was investigated to elucidate the consequences of the observed highly variable mRNA levels. Bovine ASIP is a secreted protein of 133 amino acids (UniProtKB entry: Q29414), with 77% homology to mouse ASIP and 75% to human ASIP. There are no experimentally derived data on this protein in cattle and commercial antibodies against ASIP are not available for this species. Therefore, we employed Pierce Custom Antibody Service (Thermo Scientific, Rockford, USA) to design and produce a polyclonal, bovine-specific ASIP antibody. We tested the reactivity of the antibody in Western blot and proved its specificity by blocking the binding in tissues of single anonymous cattle with the synthetic peptide which was used to raise the antibody. A specific band was detected at a higher molecular weight than theoretically expected (∼22 kDa vs. 14.8 kDa) that could be blocked in all investigated tissues (Figure S2). Additionally, several weak bands appeared at higher molecular weights, which were mostly unspecific or, if they were suppressed by the blocking peptide, suggested modifications of the ASIP protein and/or interactions with other proteins. Using a highly sensitive chemiluminescence substrate, the 22 kDa band appeared strong in different adipose tissues like SCF and intermuscular fat as well as in liver tissue, but weak in lung and skin of adult cattle. No specific band could be detected in skeletal and cardiac muscle (Figure S2). No differences in protein abundance in different tissues were found between two bulls either over-expressing ASIP mRNA or not (Figure 5).

### Cellular localization of ASIP protein in bovine tissues

**Figure 6:**
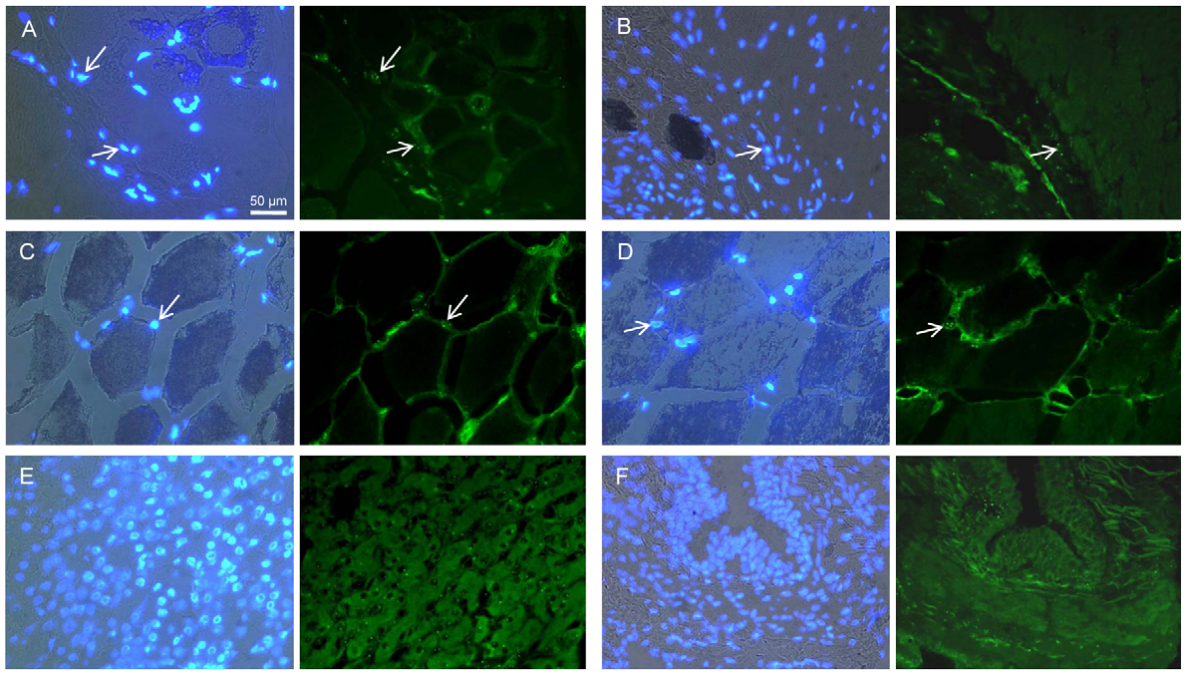
Cellular localization of ASIP protein in different bovine tissues. The left panels show the overlay of Hoechst 33258 nuclear stain with the bright-field image. In the right panels, ASIP protein is labeled with Alexa 488 (green). White arrows indicate distinct signals located at nuclei. (**A**) M. longissimus with included intramuscular fat. (**B**) cardiac muscle, (**C**) subcutaneous fat, (**D**) intermuscular fat, (**E**) liver, (**F**) lung.

Using the bovine antibody, ASIP protein was detected by immuno-fluorescence in all investigated tissue samples (Figure 6). Even in skeletal muscle with included IMF, where ASIP was not detectable by Western blot, a specific signal was observed in cells located in connective tissue as well as in intramuscular adipocytes. The signals were never attached to muscle fibers confirming that myocytes did not express ASIP. In adipocytes of intramuscular, intermuscular and subcutaneous fat, respectively, a diffuse signal was located in the cytoplasm as expected for a secreted protein. Furthermore, associated cells, located between adipocytes and without a fat vacuole, were positive for ASIP. Specific ASIP signals appeared in two distinct forms, either cytoplasmic or associated to nuclei. We observed cells which had both forms and other which had only one form of the signals. The nuclear signal was visible as a few single dots (Figure 6, white arrows). Since all of the dots were only observed directly at or in the nuclei, an artifactual signal could be ruled out. It is not yet clear whether the observed signal is attached to the nuclear membrane or is located in the nucleus itself. Preliminary Western blot analyses of proteins from cell fractions (data not shown) supported the findings that ASIP was apparent in both the cytoplasmic and nuclear fractions.

### Genomic structure of ASIP non-coding exons

As sequence variation in non-coding exons may exert effects on ASIP expression, we sequenced exons 1B, 1C and 2C. No single nucleotide polymorphism (SNP) was found in the analyzed samples from different breeds (data not shown). The current version of dbSNP (http://www.ncbi.nlm.nih.gov/projects/SNP/) lists 35 SNPs at the bovine ASIP locus, all of them located in intronic sequences. We selected two SNPs located closest to the insertion site of the L1-BT (rs109995343: 459 bp 5′ of the insertion, rs134107495: 762 bp 3′ of the insertion) and typed them in our samples to detect possible relationships with the occurrence of the transposition. None of the selected SNPs was related to the existence of the L1-BT in the individual genomes (data not shown). We then amplified and sequenced the 5′ and 3′ genomic regions, spanning the respective junctions of the LINE L1-BT to investigate its prevalence in cattle. Specific primers for the respective junctions and a primer pair specific for the genomic sequence without an insertion event were designed. As expected from the expression results, crossbred bull #4 was the only animal in this group with evidence for an L1-BT-insertion at the ASIP locus (Figure S3). However, a specific amplicon of this region without insertion of a L1-BT was obtained, too, indicating a heterozygous state at this locus. Unexpectedly, crossbred bull #1 exhibited a specific amplicon of the 5′ junction, although no expression of transcript 2C was observed in any tissue. Attempts to amplify the 3′ junction for this animal failed. Sequencing of the junctions in JB, HS, and CH confirmed the existence of exon 2C in animals expressing the respective transcript. DNA analyses in individuals of further 5 cattle breeds and crosses revealed individual cattle of different breeds with the L1-BT insertion at the bovine ASIP locus (Table S1). However, we did not observe an animal with a homozygous insertion in our sample.

### Expression of melanocortin receptors in bovine subcutaneous fat

**Figure 7:**
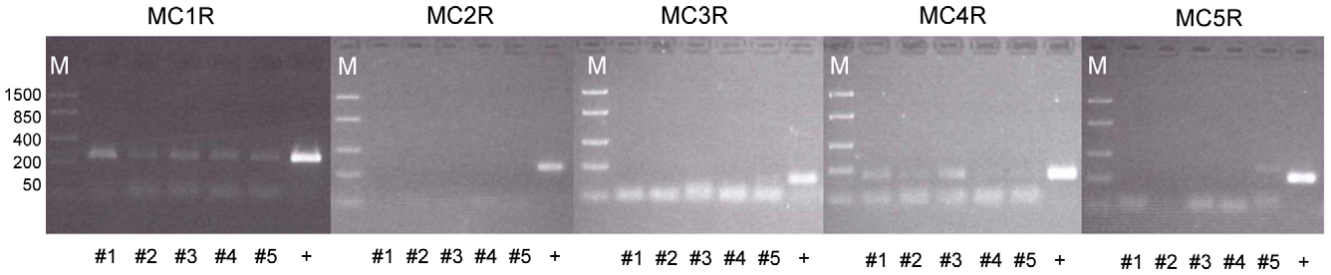
Expression of melanocortin receptors 1–5 (MCR) in subcutaneous fat (SCF) of crossbred bulls. All MCR were amplified in cDNA from SCF (40 cycles). Specific PCR products were obtained for MC1R (233 bp) and MC4R (163 bp). +: PCR product obtained in genomic DNA. M: molecular weight marker.

The expression of melanocortin receptors (MCR) 1 to 5 as potential targets for ASIP was analyzed by RT-PCR in SCF of the crossbred bulls. This included the first attempt to detect bovine MC3R mRNA because previous investigations targeted an erroneously annotated MC3R pseudogene [28], [29]. The MC2R, MC3R and MC5R were not expressed in bovine SCF, but we observed specific amplicons for MC1R and MC4R (Figure 7). A genomic PCR product was used as positive control for amplification of the intronless genes for MC1R/3R/4R/5R, and MC2R where the selected RT-PCR primers did not span both existing exons. MC1R and MC4R were expressed in SCF samples of all crossbred bulls. A MC1R-amplicon was also detected in samples from JB, HS and CH, whereas only a weak MC4R transcript was observed in some of the samples (data not shown).

### ASIP over-expression in bovine skin and coat color

Girardot et al. [12] postulated the expression of ASIP transcript 2C in skin as causal condition for brindle color phenotype in Normande cattle when at least one wild-type allele of the melanocortin receptor 1 (MC1R) is present. As we demonstrated expression of this transcript in skin of crossbred bull #4 (Figure 4) and observed the existence of this exon in different other breeds, we determined the genotypes at the MC1R locus by sequencing (Table S1). Three different alleles were observed: E (wild type, also named E^+^), E^D^ (p.Leu99Pro) and e (p.Leu104fs) [26]. In six out of 21 animals at least one wild type MC1R allele (E) was present. Among these, three JB steers and crossbred bull #4 possessed ASIP transcript 2C, thus fulfilling the requirements for a brindle coat color phenotype [12]. However, all animals were colored uniformly black with no signs of brindle hairs.

## Discussion

The agouti protein, encoded by the ASIP locus, was early assumed to play a role in lipid metabolism in human due to its strong expression in adipose tissue [10], [16], [30]. In farm animals, ASIP mRNA was recently detected in different tissues of cattle, rabbit and chicken and a potential role for fat-related traits was proposed [6], [12], [14], [31]. For this reason, we conducted a comparative, quantitative analysis of ASIP mRNA abundance in bovine adipose and further tissues and analyzed protein abundance and localization.

### Highly variable ASIP mRNA abundance in bovine tissues is caused by a single, over-expressed transcript

To elucidate a potential relationship between ASIP mRNA expression and IMF, we investigated samples of cattle with an extremely wide range of IMF deposition in M. longissimus (<2%–>39%). Observed ASIP expression differences could be assigned to individual transcripts which have earlier been identified by Northern blot in different bovine tissues [12]. A transcript recruiting non-coding exon 1B, located 60.3 kb upstream the three coding exons, was identified as responsible for a basal ASIP expression in IMF and SCF. However, this transcript was similarly expressed in breeds with different IMF deposition. Elevated ASIP expression observed in our study was solely due to the abundance of a L1-BT-derived, alternative transcript (2C).

Our initial hypothesis of a direct relationship between ASIP mRNA abundance and IMF deposition in M. longissimus was disproved since JB and single CH and CB animals over-expressed transcript 2C while displaying extremely different IMF contents,. Thus, our results underline the necessity to analyze the transcripts separately in order to avoid misleading conclusions regarding ASIP mRNA expression.

The ASIP expression pattern in heart, liver and lung was similar to that observed in adipose tissue. The basal expression of ASIP transcript 1B was complemented by over-expression of transcript 2C in single animals. In heart muscle, we could not detect specific transcript 1A described by Girardot et al. [12]. The limited reproducibility of ASIP mRNA expression in liver was similar to results obtained by other groups. Girardot et al. [12] detected a weak band with Northern blot, whereas Graphodatskaya et al. [13] could not amplify ASIP cDNA in liver samples of different bovine breeds. The results on ASIP protein expression however, indicated a stable expression of ASIP in liver tissue, too.

In contrast to the former tissues, there are up to four different transcripts expressed in bovine skin. All animals expressed skin-specific ASIP transcript 1C and ubiquitous transcript 1B, that was not described before in skin. Additionally, we could amplify the transcripts 1C2C and 2C in skin of one crossbred bull confirming results obtained earlier by Girardot et al. [6], [12].

As expected, ASIP is expressed in different bovine tissues. This is similar to observations in human but different from that in mouse where an expression outside skin is ectopic [10], [30].

### ASIP protein is expressed in different amounts in bovine tissues without close relationship to mRNA levels

We detected ASIP protein in bovine adipose tissues as well as in liver and to a much lower amount in heart, lung and skin. A specific band - strongly suppressed by the blocking peptide - with considerably higher molecular weight was detected in heart and another one in liver additionally to the expected band. It is possible that these bands are caused by ASIP attached to potential target molecules being different in heart and liver.

In the protein fraction from M. longissimus including IMF no ASIP protein expression was observed despite a comparably high mRNA abundance and high protein amounts in dissected IMF. This may be due to the low content of IMF-derived proteins among muscle proteins and the necessary high dilution of the muscle protein sample to guarantee similar protein amounts in electrophoresis like in other tissues. The low IMF content in the samples analyzed here may have led to a dilution of the IMF-derived proteins below the detection limit for ASIP protein.

Subsequent comparison of samples from two crossbred bulls with and without over-expression of mRNA transcript 2C revealed a similar pattern of protein expression in different tissues as described above and no obvious effect of transcript 2C on protein abundance. The functionality of this transcript 2C however, was proven by linking its expression in skin to a distinct phenotype - the brindled coat color in Normande cattle [12]. All animals investigated in that study were homozygous for the L1-BT insertion at the ASIP locus thus expressing two copies of the ASIP transcript 2C. One can speculate that this condition at the ASIP locus may be necessary to exert phenotypic effects. This could explain the obvious discrepancy between the highly up-regulated expression of transcript 2C and the unchanged protein levels in the respective tissues in this study.

### ASIP protein is located in cytoplasm and at nuclei in different cell types

Detection of ASIP protein by immuno-histochemistry in the tissues was largely consistent with the results obtained by Western blot. Specific fluorescence signals were observed in all investigated tissues indicating that different cell types are able to synthesize and probably secrete ASIP. It remains to be elucidated, whether non-adipocyte cells in adipose tissue secrete ASIP and affect the metabolism of adipocytes by paracrine mechanisms or whether adipocytes are affected by their endogenous ASIP production in an autocrine manner as suggested by Smith et al. [19]. The potential effects of ASIP secreted from IMF inclusions on the surrounding muscle cells could be an interesting target for future research.

ASIP expression in human internal organs was observed at mRNA level almost two decades ago [10], [32]. However up to now, neither results are available on protein expression in these tissues nor experimental evidence exists for ASIP function in heart, liver, lung or other organs. Protein expression and cellular localization of ASIP was only investigated in murine skin so far [17], [33]. Our results on cellular localization of ASIP in bovine tissues provide a basis for more detailed studies on the specific ASIP function in heterogeneous tissues composed of a variety of different cell types in all species with a widespread ASIP expression outside of skin.

Although we could not establish a link between ASIP mRNA or protein abundance and fatness traits in our study, the expression in different adipose tissue depots and organs supports a physiological function for ASIP outside melanogenesis in cattle.

### Non-coding exons of bovine ASIP are not polymorphic and L1-BT-derived exon 2C is widespread in bovine breeds

Girardot et al. [12] identified six non-coding exons forming five different transcripts in cattle. Four of these five transcripts could be detected in our study. ASIP transcripts, containing different non-coding exons located several kb upstream the first coding exon, were described in many species [6], [34]–[40]. In contrast to the highly conserved coding exons, those are species specific with less conserved sequences and different locations relative to the respective ASIP first coding exon [41]. In mice, they facilitate specificity of ASIP expression in melanocytes regarding time and body region (reviewed by Siracusa [42]). In other species including man, several non-synonymous SNP have been identified. SNP in the ASIP codons exert effects on coat and plumage color in mice, sheep, rabbit, horse and quail, respectively [5], [31], [43]–[45]. On the other hand, no clear relationship between coding SNP and skin pigmentation was found in human, pig and goat [38], [46]–[48]. Comparative sequencing of the non-coding exons 1B, 1C and 2C in parts of our sample revealed no variation. Together with results of Royo et al. [49], who did not find any SNP in the ASIP coding exons in a panel of 9 cattle breeds, this invariability defines a unique property of the bovine ASIP locus.

Bovine exon 2C is part of the internal promoter of an L1-BT retrotransposon inserted between non-coding exon 1C and coding exon 2 [12]. The transcript 2C is likely to be functional as deduced from the unchanged open reading frame. The amplification of transcripts 2C and 1B revealed Cp differing by 7 to 8 cycles. Although a direct comparison of the expression values of different transcripts is not valid in general, this observation strengthens evidence for a remarkable over-expression of transcript 2C in bovine tissues as was observed by Girardot et al. [12] using Northern blot. Initially thought to be specific to particular French breeds, our study provides evidence for a wide spread occurrence of this L1-BT insertion by detecting it in further five cattle breeds. Since these breeds strongly differ in coat color as well as in performance traits, the functional consequences of this retrotransposition remain obscure. LINE L1 repeats are the most abundant LINE type in the bovine genome. The bovine LINE L1 elements are thought to be more active in the bovine genome than in other mammalian species [50]. Insertion of transposable elements at the ASIP locus was described in mice and only recently in dogs [40], [51]. This makes a mutation hotspot region likely at this position. In contrast, a strong selection constraint is imposed on the ASIP coding region. ASIP is a protein with structural features shared only with a single paralogous protein, the agouti-related protein (AGRP). There are two domains in the N- and C-termini, respectively, which are highly conserved across mammalian and even vertebrate species [41].

We did not notice amplicons or sequences with indications of origin from putative different copies of the ASIP gene in our study. Analysis of the ovine and caprine ASIP loci revealed copy number variation (CNV) related to different color phenotypes [39], [52]. Recent genome wide scans for CNV in cattle however, did not detect signs for CNV at the bovine ASIP locus [53]–[56]. Nevertheless, the occurrence of CNV in the phylogenetically close related species sheep and goat does not completely rule out a similar phenomenon in bovine species.

### Melanocortin receptors 1 and 4 are expressed in bovine subcutaneous adipose tissue and may be potential targets for ASIP

It was early shown that ASIP is a potent antagonist of the MC1R explaining its role in melanogenesis. Furthermore, MC4R was antagonized in a similar manner, thus indicating a potential role of ASIP outside melanogenesis [3]. However, subsequent studies on interactions of ASIP with members of the MCR family in adipose tissue resulted in partly conflicting results. All five MCR were detected in human adipose tissue [57], whereas Smith et al. [19] found only MC1R and MC2R in human SCF. In contrast, Hoch et al. [20] reported only stable expression of MC1R in human SCF with MC4R and MC5R mRNA appearing only occasionally at low levels. From the lack of MC2R expression and the stable MC1R expression they concluded that melanocortins regulate cell proliferation and inflammatory processes rather than lipolysis in human adipose tissue. Although several groups detected different members of the MCR family in a variety of bovine tissues, no data exist for adipose tissue so far [29], [58]–[60]. In this context we targeted all MCR in bovine SCF. Although we could not detect MC5R at all, our data indicate similar conditions in bovine SCF as described for human. MC1R and MC4R are expressed and thus, may be putative targets for ASIP in this tissue. In a recent study on chicken adipose tissue, MC5R was the predominating member of the MCR family and MC1R was expressed to a lower extent. MC4R could be detected only occasionally [14]. This points to differences in the expression pattern of MCR between avian and mammalian species. Consequently, bovine adipose tissue may be representative for the investigation of ASIP effects in adipose tissue in non-rodent, mammalian species.

### Expression of ASIP transcript 2C from a single allele in skin does not cause brindle coat color in cattle with MC1R wild type alleles

Expression of ASIP transcript 2C was identified as causal factor for brindle coat color in Normande cattle, when a wild-type allele at the MC1R was present [12]. Although coat color was not a primary target in our study, we tested this hypothesis. We observed 4 animals in our study with the postulated condition for brindle coat color. However, none of them displayed a brindle-like coat color pattern. This clearly disproves the assumption of Girardot et al. [12]. Either a homozygous status for the insertion of the L1-BT is required, which was not observed in the animals of our study, or additional factors may be necessary to cause the brindle coat color in cattle.

### Conclusion

Our results provide evidence for a widespread expression of ASIP in bovine tissues at mRNA and protein level. Previously described transcripts of the bovine ASIP gene contribute to a different extent to the observed total mRNA amount. These differences, however, are not reflected by different ASIP protein abundance and are not related to the intramuscular fat content in cattle. Furthermore, we could show that ASIP protein expression in heterogeneous tissue like skeletal muscle is derived from adipocytes and non-adipose cells, but not from myocytes. The results on subcellular localization of ASIP indicate that beside the expected cytoplasmic presence, nucleus derived signals exist, which may be important for yet unknown functions of ASIP. Summarizing, our findings provide a basis for a more detailed investigation of the action of ASIP in peripheral tissues of non-rodent, mammalian species.

## Supporting Information

Figure S1 **ASIP mRNA abundance (all transcripts) and abundance of transcript 1B in bovine subcutaneous fat (SCF).** Bars represent means of fold changes compared to Holstein (n = 5) with 95% confidence interval, marked by vertical lines. Different letters indicate significant differences to Holstein within tissue (p<0.05). Number of samples: Japanese Black (n = 6), Charolais (n = 6), Crossbred Bulls (n = 5).
(PDF)

Figure S2 **ASIP protein expression in different bovine tissues.** Chemiluminescence detection of ASIP by Western blotting of 40 µg protein of the respective tissues. Lanes 1 and 10: molecular weight marker, 2: subcutaneous fat, 3: intermuscular fat, 4: M. longissimus, 5: heart, 6: liver, 7: lung, 8: skin (white), 9: skin (black) (**A**) Antibody against bovine ASIP 1∶10,000; (**B**) Antibody against bovine ASIP blocked with the antigen peptide. The specific bands are framed.
(PDF)

Figure S3 **Detection of the L1-BT insertion at the bovine ASIP locus in crossbred bulls.** Specific PCR products of 419 bp and 399 bp represent the genomic 5′- (**A**) and 3′- (**B**) junctions of the L1-BT. A PCR product of 430 bp spans the genomic region without insertion (**C**). Sample #4 is heterozygous for the insertion. Sample #1 revealed a specific amplicon for the 5′-junction but not for the 3′-junction.
(PDF)

Table S1 **L1-BT insertion at the ASIP locus, expression of transcript 2C and genotypes at the coat color locus MC1R in different cattle breeds and crosses.**
(PDF)

Table S2 **Primers sequences and characteristics.**
(PDF)
