# Supplementary figures and images for: Agouti Revisited: Transcript Quantification of the ASIP Gene in Bovine Tissues Related to Protein Expression and Localization
Source: PLoS One. 2012 Apr 17;7(4):e35282. doi: 10.1371/journal.pone.0035282 (PMC3328439; doi:10.1371/journal.pone.0035282)

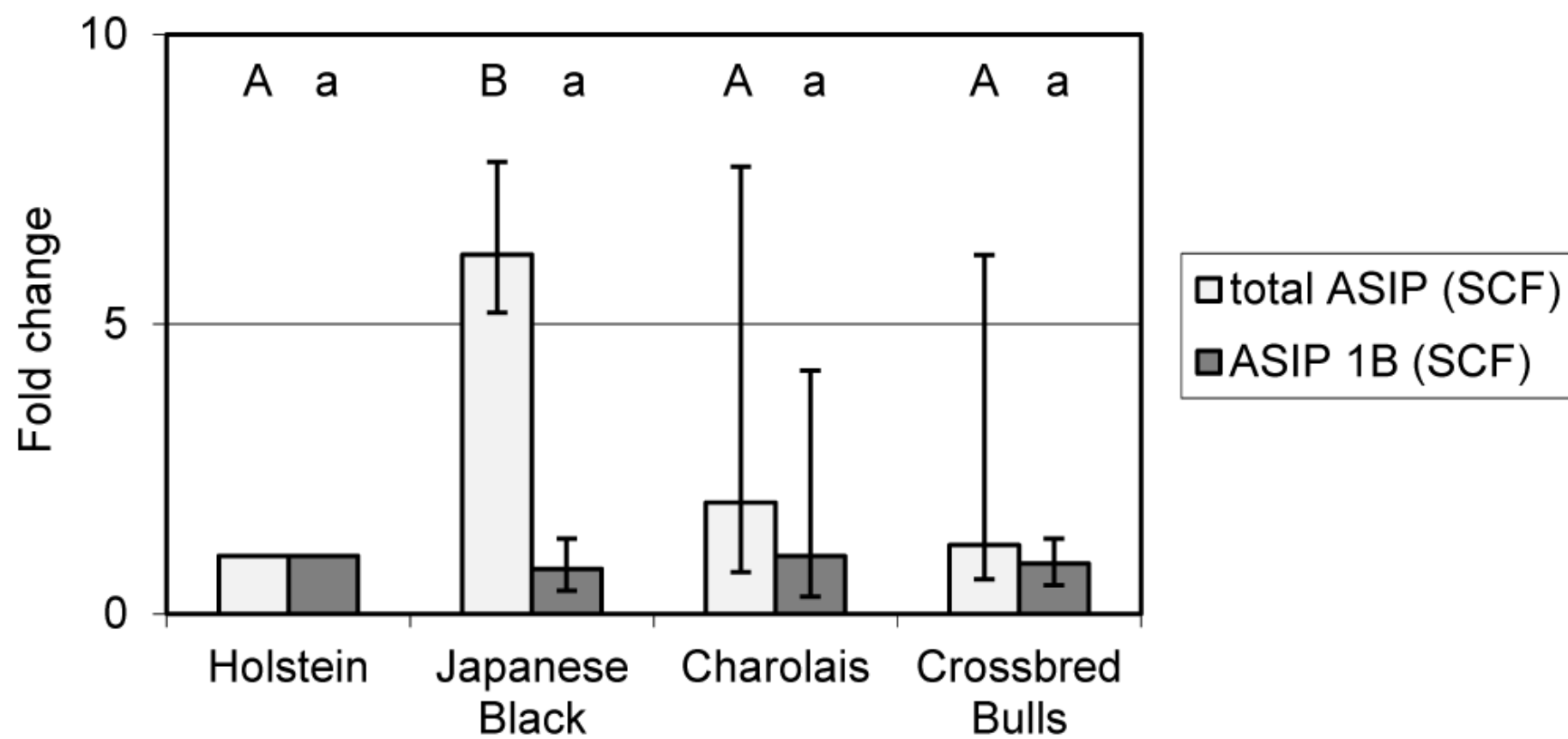

Supplement: Figure S1 — ASIP mRNA abundance (all transcripts) and abundance of transcript 1B in bovine subcutaneous fat (SCF). Bars represent means of fold changes compared to Holstein (n = 5) with 95% confidence interval, marked by vertical lines. Different letters indicate significant differences to Holstein within tissue (p<0.05). Number of samples: Japanese Black (n = 6), Charolais (n = 6), Crossbred Bulls (n = 5). (PDF) [file pone.0035282.s001.pdf]

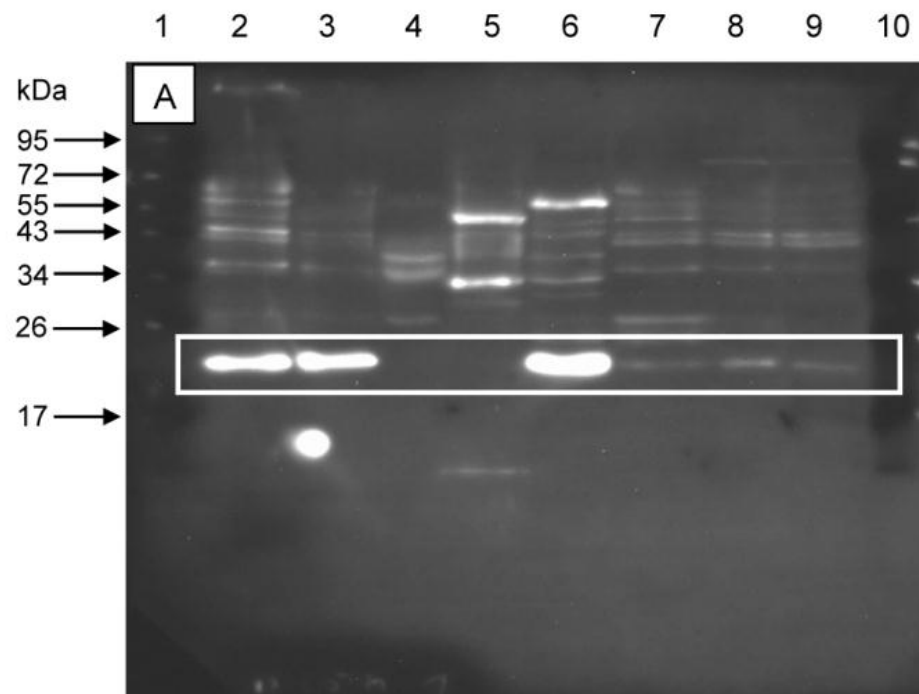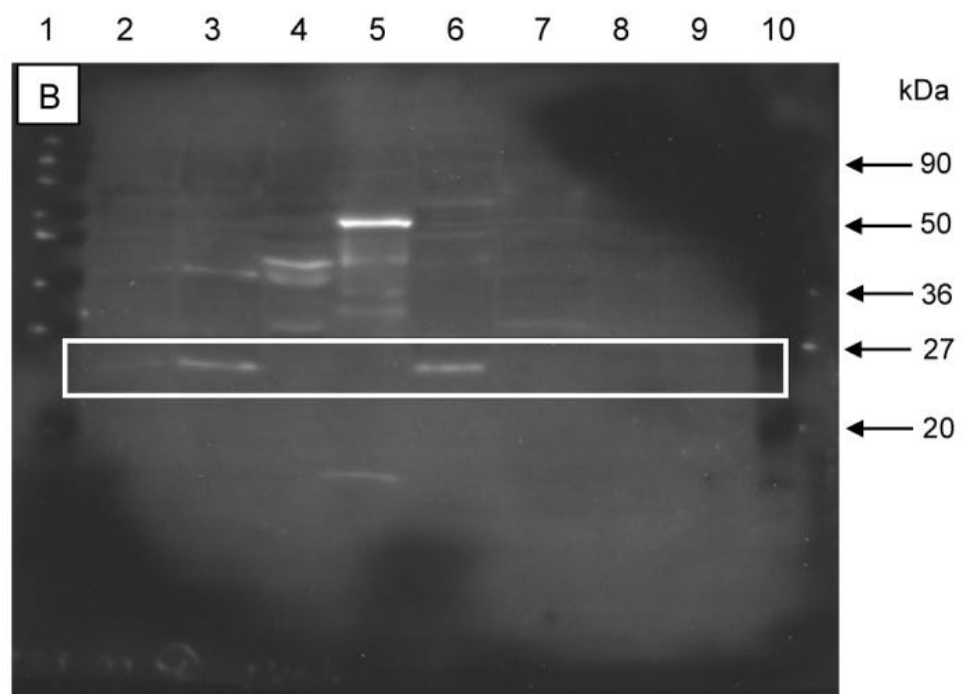

Supplement: Figure S2 — ASIP protein expression in different bovine tissues. Chemiluminescence detection of ASIP by Western blotting of 40 µg protein of the respective tissues. Lanes 1 and 10: molecular weight marker, 2: subcutaneous fat, 3: intermuscular fat, 4: M. longissimus, 5: heart, 6: liver, 7: lung, 8: skin (white), 9: skin (black) (A) Antibody against bovine ASIP 1∶10,000; (B) Antibody against bovine ASIP blocked with the antigen peptide. The specific bands are framed. (PDF) [file pone.0035282.s002.pdf]

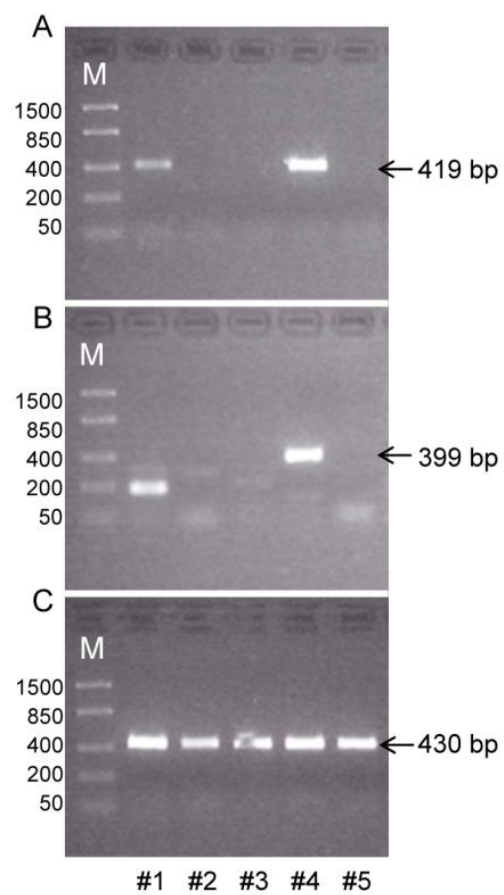

Supplement: Figure S3 — Detection of the L1-BT insertion at the bovine ASIP locus in crossbred bulls. Specific PCR products of 419 bp and 399 bp represent the genomic 5′- (A) and 3′- (B) junctions of the L1-BT. A PCR product of 430 bp spans the genomic region without insertion (C). Sample #4 is heterozygous for the insertion. Sample #1 revealed a specific amplicon for the 5′-junction but not for the 3′-junction. (PDF) [file pone.0035282.s003.pdf]
