# Supplementary material for: Agouti Revisited: Transcript Quantification of the ASIP Gene in Bovine Tissues Related to Protein Expression and Localization
Source: PLoS One. 2012 Apr 17;7(4):e35282. doi: 10.1371/journal.pone.0035282 (PMC3328439; doi:10.1371/journal.pone.0035282)
Supplement: Table S1 — L1-BT insertion at the ASIP locus, expression of transcript 2C and genotypes at the coat color locus MC1R in different cattle breeds and crosses. (PDF) [file pone.0035282.s004.pdf]

Table S1. L1-BT insertion at the ASIP locus, expression of transcript 2C and genotypes at the coat color locus MC1R in different cattle breeds and crosses.

Breed/Cross	Sample	L1-BT insertion		MC1R genotype[2]
		genomic detection	expression 2C	
Holstein	#1	no	no	E^D/E^D
	#2	no	no	E^D/E^D
	#3	no	no	E^D/E^D
	#4	no	no	E^D/E^D
	#5	no	no	E^D/E^D
Japanese Black	#1	heterozygous	yes	E/E[3]
	#2	heterozygous	yes	E^D/E^D
	#3	heterozygous	yes	E/E[3]
	#4	heterozygous	yes	E^D/E^D
	#5	heterozygous	yes	E/E[3]
	#6	heterozygous	yes	E^D/E^D
Charolais	#1	no	no	E^D/e
	#2	heterozygous	yes	E^D/e
	#3	no	no	E^D/e
	#4	no	no	e/e
	#5	heterozygous	yes	E^D/e
	#6	no	no	E^D/e
Crossbred (Jap. Black x diverse)	#1	no[1]	no	n/d
	#2	no	no	E/E^D
	#3	no	no	E/E
	#4	heterozygous	yes	E/E[3]
	#5	no	no	E^D/e
Charolais x Holstein (F_2-offspring)	#1	no		
	#2	no		
	#3	no	n/d	n/d
	#4	no		
	#5	no		
Aberdeen Angus	#1	heterozygous		
	#2	no		
	#3	heterozygous	n/d	n/d
	#4	heterozygous		
	#5	heterozygous		
German Black and White	#1	no		
	#2	no		
	#3	no	n/d	n/d
	#4	no		
	#5	no		
Galloway	#1	heterozygous		
	#2	heterozygous		
	#3	no	n/d	n/d
	#4	heterozygous		
	#5	heterozygous		
	#6	no		
White Blue Belgian	#1	no	n/d	n/d
	#2	no		

n/d: not determined

1: A specific amplicon was received for the 5’ junction, but no 3’ junction and no expression of transcript 2C was observed.

2: MC1R genotypes according to [26]; E: wild type; E^D and e: mutant alleles

3: These animals possessed the proposed genetic requirements for brindle coat color (at least one MC1R wild type allele and expression of L1-BT-derived ASIP transcript 2C [12]) but did not display the phenotype.
