# Supplementary material for: Agouti Revisited: Transcript Quantification of the ASIP Gene in Bovine Tissues Related to Protein Expression and Localization
Source: PLoS One. 2012 Apr 17;7(4):e35282. doi: 10.1371/journal.pone.0035282 (PMC3328439; doi:10.1371/journal.pone.0035282)
Supplement: Table S2 — Primers sequences and characteristics. (PDF) [file pone.0035282.s005.pdf]

Table S2. Primers sequences and characteristics

Primer name	Sequence (5’-3’)	GenBank acc. no. Position	Amplicon length (bp)	T_a (°C)	Use/Remarks
**ASIP total**		GK000013.2[1]			
for	TACCTTGCTGGTCTGCCTGT	69,449 - 69,468	181	60	qPCR, all ASIP transcripts
rev	CTTTTCCGCTTCATTTCTGC	70,944 - 70,925			
**ASIP Ex2C**		GK000013.2[1]			
for	AAAAGCAACCCAACTCTTCG	48,326 - 48,345	244	60	qPCR, ASIP transcript 2C
rev	CTTTTCCGCTTCATTTCTGC	70,944 - 70,925			
**ASIP Ex1B**		GK000013.2[1]			
for	TTTGATGAGCAAGGGTTGTG	2,312 - 2,331	157	60	qPCR, ASIP transcript 1B
rev	AGTGTTGGAGAGTGAGGACCA	2,468 - 2,448			
**UXT**[2]		NM_001037471			
for	TCATGGCGACGCCCCCTAAAC	30-50	70	60	qPCR, reference gene
rev	AAAGCCTCGTAGCGCAGCACT	99-79			
**B2M**[3]		NM_173893			
for	TGGGTTCCATCCACCCCAGATTGA	181-204	237	60	qPCR, reference gene
rev	TGTTCAAATCTCGATGGTGCTGCT	417-394			
**ASIP Ex1A**		GK000013.2[1]			
for	CAGTTGACAGGCAAATCACG	62 - 81	185	60	RT-PCR, heart-specific transcript 1A (qualitative)
rev	GGCTGTAGGCAGTGAGGAAG	69,489 - 69,470			no RT-PCR product obtained in this study
**ASIP Ex1B**		GK000013.2[1]			
for	TTTGATGAGCAAGGGTTGTG	2,312 - 2,331	562	60	RT-PCR, transcript 1B (qualitative)
rev	GGCTGTAGGCAGTGAGGAAG	69,489 - 69,470			
**ASIP Ex1C**		GK000013.2[1]			
for	GCATAAGGACAGGGAAGACG	39,658 - 39,677	132	64	RT-PCR, skin-specific transcript 1C (qualitative)
rev	GGCTGTAGGCAGTGAGGAAG	69,489 - 69,470			
**ASIP 5junc**		GK000013.2[1]			
for	AAATCAACATCTCGGCTTGG	47,911 - 47,930	419	62	PCR, genomic DNA, 5’-junction of L1-BT
rev	CTTTTCTGGGTGCCTGATGT	48,330 - 48,311			
**ASIP 3junc**		GK000013.2[1]			
for	GAAACGAGTTGCCAGTCCAG	54,600 - 54,619	399	63	PCR, genomic DNA, 3’-junction of L1-BT
rev	AAAAGGAAAGTGCGGAGGAG	54,998 - 54,979			
**ASIP no_ins**		GK000013.2[1]			
for	AAATCAACATCTCGGCTTGG	47,911 - 47,930	430	62	PCR, genomic DNA, no insertion of L1-BT
rev	AAAAGGAAAGTGCGGAGGAG	54,998 - 54,979			

Table S2. (continued)

Primer name	Sequence (5'-3')	GenBank acc. no. Position	Amplicon length (bp)	T_a (°C)	Use/Remarks
**ASIP gap**		GK000013.2[1]			
for	GGCAGGAGGATTCTTTACCC	32,857 – 32,876	312 (177+135)	60	PCR, genomic DNA, closing a gap of 135 bp
rev	AATGGACAGGGAGGCCTAGT	33,014 – 33,033			
**ASIP SNP1**		GK000013.2[1]			
for	TCATGTGGCATAGCCAAAAA	47,556 – 47,575	375	60	PCR, genomic DNA, SNP rs109994353
rev	CCAAGCCGAGATGTTGATTT	47,911 – 47,921			
**ASIP SNP2**		GK000013.2[1]			
for	TGGGTGCATATCTGGTCCTT	55,518 – 55,537	351	60	PCR, genomic DNA, SNP rs134107495
rev	AGAAGGGGACGACAGAGGAT	55,849 – 55,868			
**MC1R**		NM_174108			
for	CTGCACTCCCCCATGTACTA	488 - 507	233	61	RT-PCR (qualitative);
rev	ATGGAGATGTAGCGGTCCAC	720 – 701			DNA-sequencing (MC1R genotypes)
**MC2R**		NM_174109.2			
for	GATGTGGTGGACTCCCTGTT	435 - 454	256	60	RT-PCR (qualitative)
rev	GTCATGATGCGGTGGTACTG	553 - 534			
**MC3R**		XM_002692426			
for	CCCCTTCTTCCTCCATCTTG	768 - 787	121	60	RT-PCR (qualitative)
rev	CGGGTCGATGACGGAGTTA	888 - 870			
**MC4R**		NM_174110.1			
for	CTGATCGGGGTCTTTGTTGT	961 - 980	163	60	RT-PCR (qualitative)
rev	GGGCATAAATCAGAGGGTCA	1,123 – 1,104			
**MC5R**		NM_001015542.1			
for	GGACCGCTATGTCACCATCT	414 - 433	220	60	RT-PCR (qualitative)
rev	CAGGAGGAACATGTGGGTGT	633 - 614			

Forward and reverse primers given in the table were used for sequencing the respective amplicons.
[1]: Nucleotide positions refer to partial sequence of GK000013.2 starting at nt 64,165,041 (i.e. nt 1 in this table = nt 64,165,041).
[2]: UXT (ubiquitously-expressed transcript) was tested on suitability as reference gene in bovine adipose tissues (Hiller, unpublished).
[3]: B2M (beta-2-microglobulin) was tested on suitability as reference gene in bovine adipose tissues (Hiller et al. [24]).
